# Supplementary material for: BRCA1 Intragenic Duplication Combined with a Likely Pathogenic TP53 Variant in a Patient with Triple-Negative Breast Cancer: Clinical Risk and Management
Source: Int J Mol Sci. 2024 Jun 6;25(11):6274. doi: 10.3390/ijms25116274 (PMC11173113; doi:10.3390/ijms25116274)
Supplement: Supplementary file 1 [file ijms-25-06274-s001.zip › ijms-2993500-supplementary.pdf]

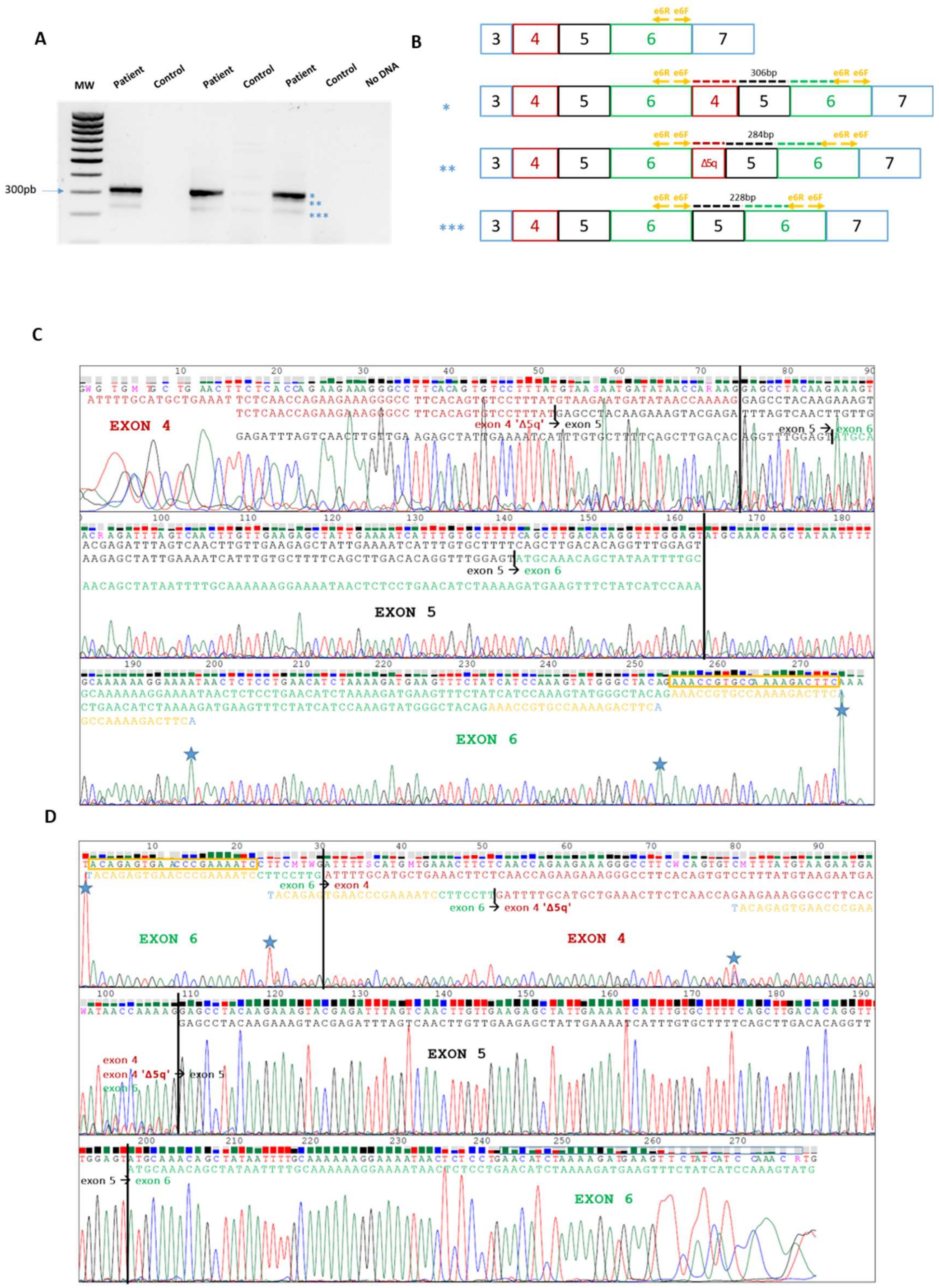

Figure S1. Sanger sequencing following RT-PCR. (A) Agarose gel electrophoresis of the RT-PCR, (*) indicate which exon scheme they are associated with in figure B (B) Schematic partial representation of the *BRCA1* mRNA, WT and with the duplication of exons 4-6. Primers used for the duplication specific RT-PCR are indicated by horizontal orange arrows, with the expected PCR product in colored dashed lines. Identified alternative transcripts are also shown. (C and D) Electropherograms using e6F (C) or e6R

(D, reversed orientation) primer for Sanger sequencing. In visual reading of double/triple peaks, nucleotides related to exons 4, 5, 6 and primer sequence are colored in red, black, green and orange, respectively. Vertical black lines indicate exon-exon junctions, which are explicited for alternative transcripts. Blue stars highlight artefactual A/T nucleotide added at the end of the sequence.
